# Personalized neural responses to self and filial reward: Functional and structural responses to motivational cues

**DOI:** 10.3934/Neuroscience.2025029

**Published:** 2025-11-19

**Authors:** Siti Mariam Roslan, Siti Hajar Zabri, Nur Ayunie Ayob, Aini Ismafairus Abd Hamid, Nur Hartini Mohd Taib, Rahimah Zakaria, Sofina Tamam, Hazim Omar, Alwani Liyana Ahmad, Wan Mohd Zahiruddin Wan Mohammad, Aleya Aziz Marzuki, Asma Hayati Ahmad

**Affiliations:** 1 School of Medical Sciences, Health Campus, Universiti Sains Malaysia, 16150 Kubang Kerian, Kelantan, Malaysia; 2 Brain & Behaviour Cluster, School of Medical Sciences, Universiti Sains Malaysia, 16150 Kubang Kerian, Kelantan, Malaysia; 3 Faculty of Science and Technology, Universiti Sains Islam Malaysia, 71800 Nilai, Negeri Sembilan, Malaysia; 4 Department for Psychiatry and Psychotherapy, University of Tübingen, Tübingen, Germany

**Keywords:** reward, cues, filial, fMRI, dMRI

## Abstract

Understanding how individuals interact with reward-related cues in their environment provides insight into the neural mechanisms underlying motivation and personalized behavior. While monetary rewards for self are well studied, the neural basis of socially relevant rewards—such as filial reward—remains less understood. This study investigated functional and structural brain responses to reward gained from a cognitive task performance for self or for parents (filial) using functional MRI (fMRI) and diffusion MRI (dMRI) in young adults, reflecting personalized interaction with environmental cues. Thirty-two healthy young adults (17 males, mean age = 23 ± 1 years) performed a 2-back working memory task cued for reward conditions for self and for parents during fMRI scanning, followed by dMRI acquisition. Participants were categorized based on the reward condition in which they showed the highest score in task performance. Self-reported reward responsiveness scores were also collected. Random-effects fMRI analysis revealed activation of the putamen in the self-reward condition, more than in the filial reward condition. Using this region as a seed, probabilistic tractography was conducted to compute connection probability indices (CPI) to key target areas: anterior cingulate cortex (ACC), posterior cingulate cortex (PCC), dorsolateral and ventrolateral prefrontal cortices, anterior/posterior insula, and amygdala. The nucleus accumbens (NAcc) was included as a comparative seed. While the cue for self-reward elicited activation in the reward area putamen, with higher white matter connectivity from the right putamen to the ACC, a cue for filial reward significantly activated the right insula. Lateralization to the right insula was also seen in the structural connectivity to NAcc in the filial group. Filial reward also displayed a positive relationship between white matter connectivity of left NAcc to PCC with reward responsiveness. These results demonstrate individualized neural responses shaped by the self and social relevance of the reward.

## 1. Introduction

A recent social experiment that gained widespread attention highlighted how children often choose gifts for their parents, foregoing rewards for themselves [1]. Expressions of altruistic behavior, particularly within dynamics, have been studied through neuroimaging techniques, such as functional magnetic resonance imaging (fMRI) [2]–[5] and, to a lesser extent, diffusion MRI (dMRI) [6],[7]. Altruism denotes giving behavior toward others, encompassing a more general scope, while altruism toward parents is specifically a filial act—the word *filial* addressing the relation of a child or offspring. Meta-analyses on social altruism have shown that giving to others leads to changes in neural expression [3],[8]. However, research on the neurobiological basis of different perspectives of reward, particularly of filial relationships, remains limited. To our knowledge, no known functional or structural neuroimaging studies have examined filial altruism, prioritizing giving to parents over self.

With the advent of extreme technological advancement, there are many uncertainties regarding its transformation into positive social and economic outcomes. Current economic situations, automation, and social media all play significant roles in shaping the youth of today. Studies have shown that reward motivates. While it is well-known that giving rewards drives performance in tasks such as working memory, it is not clear how different forms of reward affect performance and how choices are made between the forms of reward offered.

The brain's reward network plays a critical role in altruistic behavior [3]. The network, which regulates human emotions like pleasure and happiness, also controls complex cognitive processes, such as decision-making and emotion regulation [9]. Rewards convey essential information that impacts choices between different courses of action. Neuroimaging, especially fMRI and dMRI, provides valuable insight into reward processing [10]–[13]. fMRI measures brain activities by monitoring blood flow changes [14], while dMRI reveals structural information about white matter connectivity [15], essential for efficient communication among reward-related brain regions.

Animal studies and human neuroimaging studies have established the importance of the striatum in processing reward-related information and mediating goal-directed behavior [16],[17]. Positioned as the gateway to the basal ganglia, the striatum integrates cortical and dopaminergic inputs, influencing both motor and cognitive behaviors [17]–[19]. Within the striatum, the putamen and nucleus accumbens (NAcc) mediate reward anticipation and motivational processes [20]. These regions, closely associated with dopamine, play distinct roles, whereby the putamen is more action-oriented [21] when responding to specific stimuli, and NAcc is integral to learning and motivation, independent of stimulus type [22]. Structurally, the putamen receives input primarily from the dorsolateral prefrontal cortex (DLPFC), and motor areas [23],[24], whereas the NAcc has stronger connections with the orbitofrontal cortex (OFC), ventromedial prefrontal cortex (VMPFC), ventrolateral prefrontal cortex (VLPFC), amygdala, and insula [17],[25].

Age-related changes in reward processing have also been observed, with reward networks evolving from the insula and striatum in children to a more extended network in adulthood, involving the cingulate, insula, basal ganglia, and thalamus [26]. Research indicates that parental warmth is associated with specific neural responses in offspring within reward and emotion regulation networks [27], particularly the medial prefrontal cortex (mPFC), striatum, and amygdala [28]. Guerra et al. [29] compared filial and romantic love using an electroencephalogram (EEG) and found that although both elicited intense positive emotional reactions, they differed in measures of valence, arousal, and dominance. One brain area that has often appeared in studies involving social and familial (including parental) interaction is the posterior cingulate cortex (PCC) [30]–[32]. Being part of the default mode network (DMN), the PCC is known for its involvement in attention, autobiographical memory [30], and conscious awareness [33]. Vila et al. [32] demonstrated that familiar faces activate the PCC, while Tamam et al. [33] found that the presence of a loved one enhanced pain tolerance and intrinsic connectivity in the anterior-posterior-middle cingulate cortex (ACC-PCC-MCC) circuit.

Despite these insights, few studies have focused on the functional and structural connectivity of the reward networks in response to self versus parent-oriented reward preferences. Our study aimed to investigate how young adults respond to rewards specifically cued to benefit the self or parents (filial), based on the performance in a cognitive task. Additionally, we sought to determine whether different reward preferences influence typical neural processes and connectivity patterns in reward-related brain regions through a combination of fMRI and dMRI.

## 2. Materials and methods

### 2.1. Participants

#### 2.1.1. Recruitment

Thirty-four participants (17 males) were initially recruited among undergraduate and postgraduate students at the Health Campus of Universiti Sains Malaysia, Kelantan, Malaysia, through purposive sampling. Recruitment was conducted through public advertisements, word-of-mouth, and mailing lists. Eligible participants were university students aged 18–24 years during data collection. Of the 34 participants recruited, one was excluded from the study due to incomplete response, which prevented group categorization. Thus, the final sample comprised 33 participants (17 males, 16 females; mean age 23 ± 1). Participants received an honorarium for their participation and an additional reward based on their performance in the cognitive task.

#### 2.1.2. Ethics approval of research

All participants provided written informed consent before the study. This study protocol was approved by the Human Ethics Committee Universiti Sains Malaysia (USM/JEPeM/21100659).

#### 2.1.3. Inclusion and exclusion criteria

Participants were pre-screened via online questionnaires to ensure eligibility. Inclusion criteria required that participants could understand MRI instructions and provide written informed consent in English. Exclusion criteria included any history of medical or psychiatric disorder that could affect brain function, current use of psychotropic medication, history of inpatient psychiatric hospitalization, use of drugs affecting the central nervous system, and substance abuse. Additionally, participants were excluded if they were pregnant, claustrophobic, or had any contraindications for fMRI, such as metallic implants, stents, or clips.

### 2.2. Study design and study procedure

This cross-sectional study utilized a block-design 2-back task performed in the fMRI scanner under three reward conditions: cash, filial, and certificate. Participants were given instructions on the cues and the cognitive task to be performed during scanning. Specific cues were presented for each reward condition, informing participants that their reward would be based on their highest performance score, along with a standardized participation remuneration. The assumption was that more effort would be expended on the task under the condition that they chose to have the reward. The reward conditions were 1) cash, in which participants would receive a monetary reward for themselves, 2) filial, in which a monetary reward would be sent to the participant's parents accompanied by a letter detailing the participant's involvement in a brain imaging study, and 3) certificate, in which participants would be awarded a certificate of participation, signed by the Dean of School of Medical Sciences.

**Figure 1.**
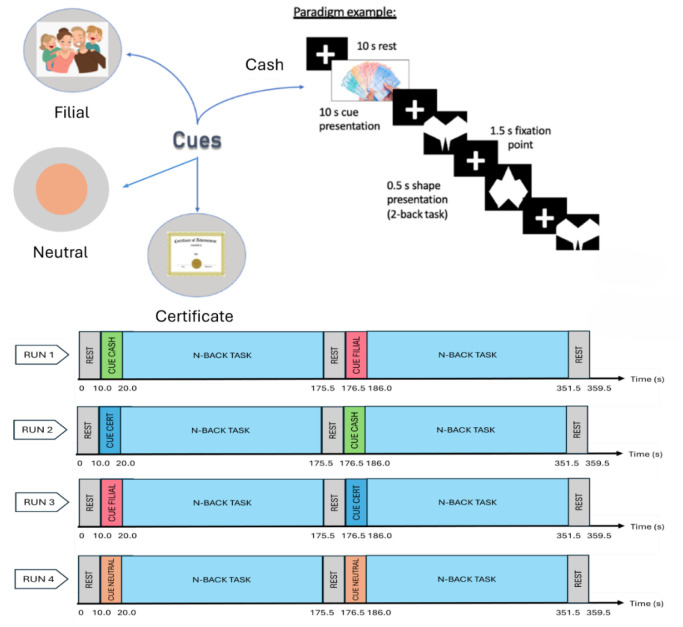
(Above) Task paradigm showing the four reward cues: neutral, cash, filial, and certificate. (Below) The experimental paradigm includes four runs of the 2-back task. Each run consists of two blocks, with each block featuring two different reward cues. Across all runs, participants completed two blocks for each cue type, allowing for balanced exposure to each reward condition.

Following each cue, participants performed the 2-back task, with cumulative scores in each condition determining their reward. Responses were classified as correct if the participant identified the correct shape within the allocated time. No feedback on response accuracy was provided during the task. A practice session of the 2-back task was conducted in a simulator room to ensure participants understood the task requirements. Visual cues for each reward condition were displayed prior to each run: a brown circle for the neutral cue, a cash icon for the cash cue, a cartoon illustration of parents for the filial cue, and a certificate icon for the certificate cue (Figure 1).

During fMRI scanning, participants completed four runs of the 2-back task. Each run included two blocks, each with two different cues. In total, participants completed two blocks for each cue, with the order of the cues pseudorandomized across participants (Figure 1). Following the fMRI scanning, participants stayed in the scanner for the dMRI scanning.

### 2.3. Questionnaires

To assess handedness, the Edinburgh Handedness Inventory [34] was administered. To evaluate participants' sensitivity to pleasant reinforcers, the reward-responsiveness questionnaire [35] was completed by participants both before and after the scanning session.

### 2.4. fMRI

#### 2.4.1. Acquisition

Functional imaging was conducted on a 3-Tesla Philips Achieva scanner equipped with a 32-channel head coil. T2*-weighted images were acquired using an echoplanar imaging (EPI) sequence with the following parameters: repetition time (TR) = 2000 ms, echo time (TE) = 30 ms, flip angle = 90°, acquisition time (TA) = 1950 ms, number of slices = 40, voxel size = 1.7 × 1.7 × 2 mm^3^, field of view (FOV) = 192 mm × 192 mm × 120 mm, number of slices = 40, interleave acquisition, no slice gap, and 180 functional volumes. The total duration of the fMRI scan was 5 min and 55 s per run. Additionally, a high-resolution anatomical image was obtained using a T1-weighted sequence (TR = 120 ms, TE = 3.4 ms, flip angle = 8°) for co-registration of functional data.

#### 2.4.2. Preprocessing

fMRI data were preprocessed using Statistical Parametric Mapping 12 (SPM12, version 6225, The Welcome Centre for Human Neuroimaging) in MATLAB R2019b (The MathWorks, Inc.). Preprocessing steps included 1) slice timing correction applied using a standard interpolation function to account for temporal offsets between slices [36],[37], 2) realignment and unwarping to correct for motion artefacts [37],[38], 3) co-registration of functional images with individual anatomical images for precise alignment [39], 4) segmentation of the anatomical images into grey matter, white matter, cerebrospinal fluid, and other tissues, which were then normalized to a standard template, 5) normalization to align all images to the Montreal Neurological Institute (MNI) space [39], using the East-Asian brain template to improve spatial accuracy, and 6) smoothing with an 8-mm full width at half maximum (FWHM) Gaussian kernel to reduce noise, enhance true activation patterns, and meet statistical analysis assumptions [40]. Motion correction was performed using SPM realignment. None of the participants exceeded 2 mm translation or 2° rotation, indicating that all data were within acceptable limits for fMRI analysis.

#### 2.4.3. Postprocessing

The first-level analysis was conducted using a general linear model (GLM) approach, with the equation y = xβ + ε [37]. Here, y represents the functional images of the reward cues, x denotes the cues, β represents the unknown parameters to be estimated, and ε includes motion-related errors, translation (x, y, z), and rotation (pitch, roll, yaw). A high-pass filter with a cutoff of 128 s was applied to remove low-frequency noise, temporal autocorrelation was modeled with an AR (1) process, and an absolute masking threshold of 0.8 was applied to restrict the analysis to voxels with sufficient signal. The model incorporated multiple conditions, i.e., cue cash, cue filial, cue certificate, and cue neutral. The design matrix was created, and model estimation computed the β values.

#### 2.4.4. Group analysis

Second-level analysis was performed using random effects analysis (RFX) to account for inter-participant variance [41]. This consecutive approach allowed broader population inferences and highlighted significant effects across participants. A full factorial design included two factors: group (cash group and filial group) and cue (cash cue, filial cue, certificate cue, and neutral cue). A two-way ANOVA was applied, identifying reward-related brain area(s) for subsequent regions of interest (ROI) analysis in the dMRI.

### 2.5. dMRI

#### 2.5.1. Acquisition

After completion of the fMRI scan, participants remained in the scanner for dMRI acquisition. Imaging parameters for the dMRI were as follows: 64 non-collinear diffusion-sensitizing gradient directions, matrix size of 128 × 128, FOV of 221 mm × 221 mm, TR/TE of 9410/96 ms, SENSE factor of 2, EPI factor of 67, b-value of 1000 s/mm^2^, and a slice thickness of 2 mm. Additionally, a single non-diffusion weighted (b0) scan was included.

#### 2.5.2. Preprocessing

Raw diffusion data underwent preprocessing to correct for artifacts, motion, and eddy currents, using tools from FMRIB Diffusion Toolbox (FDT, Oxford) [42],[43] within FMRIB Software Library (FSL). T1-weighted MRIs were processed with the Brain Extraction Tool (BET) to generate a skull-stripping mask [44], which was then applied to co-registered T2-weighted MRIs aligned to T1 image space. Skull stripping of the b0 map was also conducted before aligning it to the T2-weighted image. Next, Bayesian Estimation of Diffusion Parameters Obtained using Sampling Techniques (bedpostx) was applied, which uses Markov Chain Monte Carlo (MCMC) sampling to estimate distributions of diffusion parameters at each voxel. Automated modeling of crossing fibers was performed using bedpostx with the following parameters: number of fiber orientations per voxel = 2, weight = 1, burnin =1000, and number of jumps = 1250. Bedpostx produces a set of files ready for tractography.

#### 2.5.3. Probabilistic tractography

**Figure 2.**
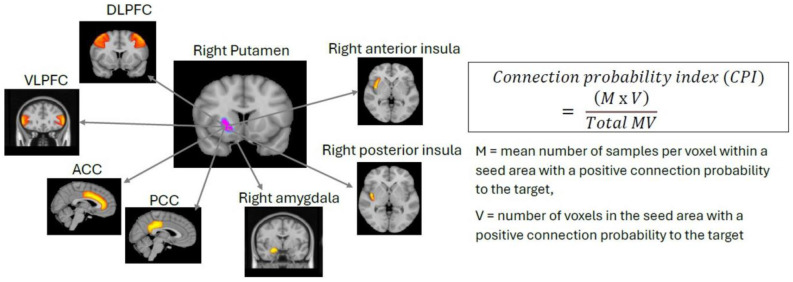
Calculation of the connection probability index (CPI). The putamen and NAcc were selected as seed regions for probabilistic tractography to determine the CPI to seven target regions: DLPFC, VLPFC, ACC, PCC, amygdala, anterior insula, and posterior insula.

Probabilistic tractography was performed to estimate white matter connectivity from the preprocessed diffusion data. Two seed regions were used; the putamen was identified from the fMRI analysis, and NAcc was chosen based on its role in reward processing literature [45],[25],[22], though it did not show activation in the fMRI analysis. Target regions included the anterior cingulate cortex (ACC), posterior cingulate cortex (PCC), ventrolateral prefrontal cortex (VLPFC), dorsolateral prefrontal cortex (DLPFC), anterior insula, posterior insula, and amygdala, all areas associated with cognitive functions relevant to this study. Seed and target regions were outlined from the Harvard-Oxford cortical and subcortical structural atlases in the FSLeyes software and thresholded accordingly. In general, the thresholding percentage for subcortical regions was higher than for cortical areas. For example, for the amygdala, the thresholding was 50%, whereas for DLPFC, it was 10%. The masks of these seed and target regions in standard space were then registered onto the standardized brain of individual subjects before running tractography. The “probabilistic fiber tracking” algorithm was employed using probtrackx in FDT, which samples from a distribution of possible fiber direction in each voxel, to simulate the diffusion process and generate multiple streamlines from each seed region [42]. For each voxel in the seed region, 5000 streamline samples were generated to establish connectivity distribution to each target. From these streamlines, the connection probability index (CPI) was calculated as the proportion of the streamlines reaching each target region from each seed [46] (see Figure 2 for CPI calculation).

### 2.6. Statistical analysis

Data were analyzed using the Statistical Package for the Social Sciences (SPSS) version 22. Repeated measures ANOVA was used to determine differences in the accuracy of the 2-back task among the groups. The Wilcoxon signed-rank test was used for the within-participants comparison of the CPI across target regions and hemispheres. Between-group comparisons of CPI percentages between the reward groups were conducted using the Mann–Whitney U test. To assess the relationship between CPI and the reward responsiveness scores, Spearman's rank correlation coefficient was applied. A significance level of p < 0.05 was set for all statistical tests.

## 3. Results

### 3.1. Grouping of participants according to 2-back scores

**Figure 3.**
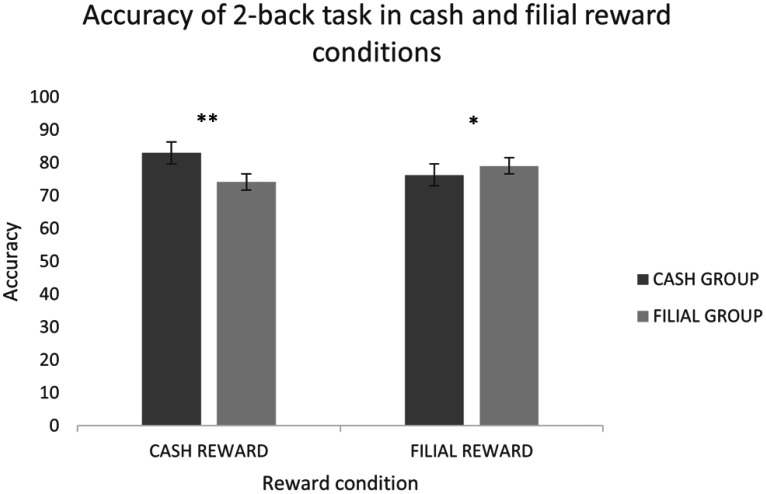
Accuracy of the 2-back task in cash and filial reward conditions. Data are mean ± SEM. *p < 0.05, **p < 0.01.

Based on the 2-back task results, the number of participants with the highest performance score across the three reward conditions was cash (n = 15), filial (n = 16), and certificate (n = 2). Participants were grouped according to the reward condition that they scored highest in. Since only two participants scored highest in the certificate reward condition, they were excluded from analysis. Repeated measures ANOVA with a within-subjects factor reward condition (two levels: cash and filial) and between-subjects factor group (2 levels: cash group and filial group) was performed to find differences in accuracy. There were significant main effects of reward condition [F(1, 29) = 5.844, p = 0.022] as well as interaction between reward condition and group [F(1, 29) = 21.30, p < 0.001]. A post hoc t-test showed significantly higher accuracy for the cash reward condition in the cash group compared to the filial group (p = 0.002), whereas in the filial reward condition, the filial group scored significantly higher compared to the cash group (p = 0.039) (Figure 3).

### 3.2. fMRI brain activation when viewing cues

**Figure 4.**
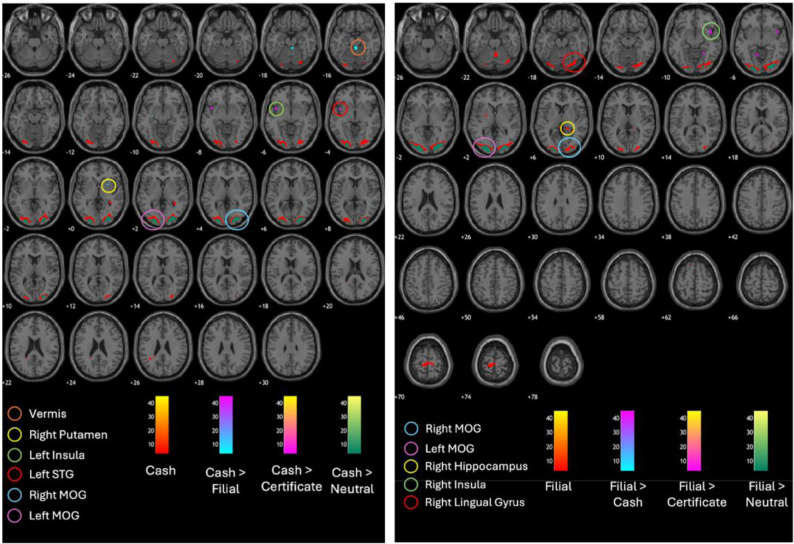
Brain activations in the cash (left) and filial groups (right) at voxel-level threshold *p_uncorrected_* < 0.001. (Abbreviations: STG = superior temporal gyrus, MOG = middle occipital gyrus).

During fMRI analysis, one additional participant was excluded due to technical errors during data collection, leaving 32 participants for analysis. RFX was performed to measure brain activation while viewing three different cues (cash, filial, and certificate), and to compare activations among the two reward groups: cash and filial (Table 1 and Figure 4).

At a strict voxel-level threshold of p < 0.05, corrected for family-wise error (FWE), no significant differences in brain activations were observed between the cash and filial groups. When applying a voxel-wise false discovery rate (FDR) correction at p < 0.05, no voxels survived the correction (FDRc = ∞). However, when using an uncorrected voxel-level threshold of p < 0.001, significant brain activations were identified across reward groups, and the corresponding FDR-corrected thresholds were reported where available.

For the cash group, the analysis was refined to a voxel-level threshold of *p_uncorrected_* < 0.001 with further adjustment at the cluster level of FDR < 0.05. Under this criterion, significant brain activations were localized to the right middle occipital gyrus (MOG) and left MOG while viewing the cash cue. In the contrast analysis of the cash group, for the contrast Cash > Filial cue, significant activations were observed in the vermis_1_2, right putamen, left putamen, right superior temporal gyrus (STG), left fusiform gyrus, and right MOG at the voxel-level threshold of *p_uncorrected_* < 0.001. When comparing Cash > Certificate cues in the cash group, activation was found in the left insula, right MOG, and right middle temporal gyrus (MTG) when thresholded at voxel-level *p_uncorrected_* < 0.001. On the other hand, when comparing Cash > Neutral cue, the brain regions activated were the right and left MOG when thresholded at voxel-level *p_uncorrected_* < 0.001 with further adjustment at the cluster level of FDR < 0.05.

**Table 1.** Brain activations associated with viewing cash, filial, certificate, and neutral cues in cash and filial groups thresholded at voxel-level p < 0.001 (uncorrected). FDR-corrected p-values are shown where applicable; n.s.: not significant or correction not applicable (FDRc = ∞).

**Reward group**	**Reward cue contrast**	**Brain activation in AAL (BA)**	**Cluster extent (voxels)**	**Maximum T-value**	**p-value (uncorr)**	**p-value (FDR-corr)**	**Peak MNI coordinates**		
							X	Y	Z
Cash	Cash	Right MOG (NA)	697	8.83	0.000	0.000	25	−90	5
		Left MOG (NA)	776	8.69	0.000	0.000	−21	−98	2
	Cash > Filial	NA (NA)	6	4.06	0.000	n.s.	−25	−14	−10
		Vermis_1_2 (NA)	36	3.88	0.000	n.s.	4	−37	−19
		Right putamen (NA)	9	3.52	0.000	n.s.	27	11	−1
		Left putamen (NA)	3	3.50	0.000	n.s.	−27	13	2
		Right STG (NA)	7	3.39	0.000	n.s.	44	−43	5
		Left fusiform gyrus (NA)	2	3.29	0.001	n.s.	−16	−37	−16
		Lateral ventricle (NA)	2	3.29	0.001	n.s.	−23	−45	5
		Left putamen (Putamen)	2	3.24	0.001	n.s.	−25	−9	5
		Right MOG (NA)	1	3.17	0.001	n.s.	33	−88	5
	Cash > Certificate	Left insula (BA 13)	39	3.77	0.000	n.s.	−42	8	−7
		Right MOG (NA)	14	3.40	0.000	n.s.	32	−90	5
		Right MTG (NA)	5	3.28	0.001	n.s.	45	−62	−1
		NA (NA)	1	3.22	0.001	n.s.	−32	−50	29
		NA (NA)	2	3.20	0.001	n.s.	−35	−52	−7
	Cash > Neutral	Right MOG (NA)	324	5.89	0.000	0.001	25	−90	5
		Left MOG (NA)	255	5.35	0.000	0.001	−21	−98	2
Filial	Filial	Left MOG (NA)	1026	10.33	0.000	0.000	−21	−98	2
		Right calcarine (NA)	1325	8.69	0.000	0.000	20	−97	−1
	Filial > Cash	Right PrG (NA)	7	3.54	0.000	n.s.	35	−21	68
		Right insula (BA 13)	8	3.44	0.000	n.s.	40	8	−10
	Filial > Certificate	Right insula (NA)	44	4.28	0.000	n.s.	44	8	−10
		Left cerebelum_4_5 (NA)	18	3.66	0.000	n.s.	−11	−54	−7
		Right lingual gyrus (BA 19)	10	3.48	0.000	n.s.	25	−49	−10
		Left MOG (NA)	4	3.37	0.000	n.s.	−51	−74	−1
		Right hippocampus (Pulvinar)	2	3.34	0.001	n.s.	15	−35	8
		Left parahippocampus (NA)	3	3.30	0.001	n.s.	−16	−38	−13
		Right cuneus (NA)	1	3.26	0.001	n.s.	11	−91	23
		Right SOG (NA)	1	3.22	0.001	n.s.	20	−90	23
		Left lingual gyrus (NA)	1	3.17	0.001	n.s.	−25	−61	−4
	Filial > Neutral	Left MOG (NA)	477	6.73	0.000	0.000	−21	−98	2
		Right IOG (NA)	520	4.97	0.000	0.030	23	−95	−4
Conjunction analysis (Cash = Filial)	Cash	Right calcarine (NA)	712	8.53	0.000	0.000	−20	−98	−1
		Right MOG (NA)	615	8.28	0.000	0.000	28	−90	5
	Filial	Right calcarine (NA)	357	7.06	0.000	0.000	−20	−98	−1
		Right SOG (NA)	388	6.87	0.000	0.000	21	−95	5
	Certificate	Left MOG (NA)	232	5.82	0.000	0.000	−21	−98	2
		Right SOG (NA)	301	5.79	0.000	0.000	21	−97	5
	Neutral	n.s.	n.s.	n.s.	n.s.	n.s.	n.s.	n.s.	n.s.

Note: *Abbreviations: n.s. = not significant, AAL = anatomical automatic labeling, BA = Brodman areas, MOG = middle occipital gyrus, SOG = superior occipital gyrus, PrG = precentral gyrus, STG = superior temporal gyrus, IOG = inferior occipital gyrus, MTG = middle temporal gyrus.

For the filial group, analysis at the voxel-level threshold of *p_uncorrected_* < 0.001 with further adjustment at the cluster level of FDR < 0.05 revealed significant activation in the left MOG and right calcarine gyrus while viewing the filial cue. When thresholded at voxel-level *p_uncorrected_* < 0.001, the contrast of Filial > Cash cues showed significant activation in the right precentral gyrus (PrG) and right insula. Similarly, when comparing Filial > Certificate cues, several regions were significantly activated, including the right insula, left cerebellum_4_5, right and left lingual gyrus, left MOG, right hippocampus, left parahippocampal gyrus, right cuneus, and right superior occipital gyrus (SOG). In contrast, when comparing Filial > Neutral cues, activation was observed in the left MOG and right inferior occipital gyrus (IOG) at voxel-level *p_uncorrected_* < 0.001.

A conjunction analysis was conducted to identify shared brain activations across the two groups (cash and filial) while viewing the reward cues. Using a voxel-level threshold of *p_uncorrected_* < 0.001 with further adjustment at the cluster level of FDR < 0.05, significant overlapping activations were observed in the cash cue condition within the right calcarine gyrus and right MOG. For the filial cue, overlapping activations were found in the right calcarine gyrus and right SOG. For the certificate cue, overlapping activations emerged in the left MOG and right SOG. Collectively, these findings suggest a convergent pattern of activation within occipital and visual association cortices across both groups, reflecting a shared neural network underlying the visual and attentional processing of reward-related stimuli.

### 3.3. dMRI connection probability index

Similarly, for dMRI, only cash and filial groups were compared. From the fMRI results, the putamen emerged as a significantly activated region associated with reward; hence, it was chosen as the primary seed to assess structural connectivity to seven target regions: ACC, PCC, VLPFC, DLPFC, anterior insula, posterior insula, and amygdala. Additionally, another area known for reward processing, the NAcc, although not significantly activated in the fMRI analysis, was included as a secondary seed region to evaluate its connectivity to the same target regions. The CPI was calculated using probabilistic tractography.

#### 3.3.1. Comparison between cash and filial groups

**Figure 5.**
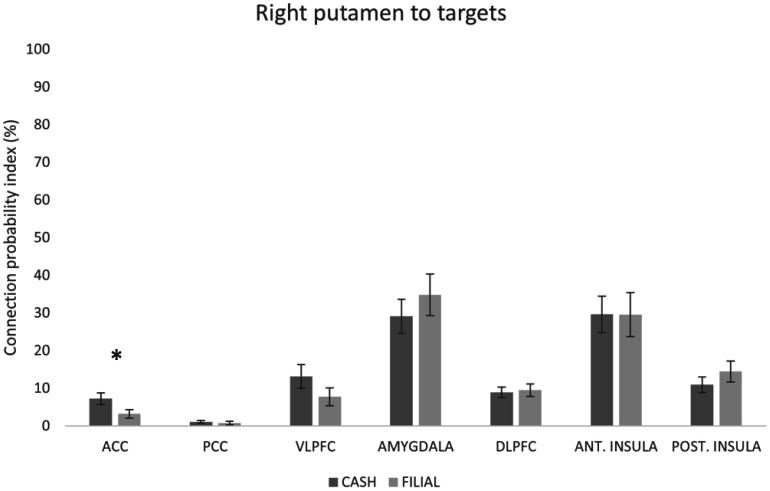
Mean CPI from right putamen to ACC, PCC, VLPFC, amygdala, DLPFC, anterior insula, and posterior insula for cash and filial groups. Data are presented as mean ± SEM. *p < 0.05. ACC = anterior cingulate cortex, PCC = posterior cingulate cortex, VLPFC = ventrolateral prefrontal cortex, DLPFC = dorsolateral prefrontal cortex.

Results of the Mann–Whitney U test indicated that the cash group showed significantly greater CPI from the right putamen to ACC than the filial group (z = −2.696, p = 0.007), suggesting stronger white matter connectivity between the right putamen to ACC in the cash group (Figure 5). No significant difference was found between the cash and filial groups in the structural connectivity between NAcc and any of the targets.

#### 3.3.2. Comparison between the left and right brain hemispheres

Comparison of CPI between the left and right brain hemispheres (Table 2) revealed distinct patterns in the cash and filial group. In the cash group, CPI was significantly higher from the left NAcc to ACC compared to the right hemisphere (Z = −2.040, p = 0.041). In addition, there was a significant difference in CPI from the putamen to DLPFC between hemispheres (Z = −2.101, p = 0.036), with the left hemisphere showing a higher CPI.

Conversely, in the filial group, significantly higher CPI was observed from the right NAcc to the posterior insula compared to the left hemisphere (Z = −2.040, p = 0.041).

**Table 2.** Comparison of median CPI between the left and right hemispheres (p < 0.05).

Group	CPI	Hemisphere	Median (IqR)	Z-statistic	p-value
Cash	NAcc to ACC	Left	2.1207 (4.36)	−2.040	0.041
		Right	0.8080 (2.55)		
Cash	Putamen to DLPFC	Left	16.8220 (8.44)	−2.101	0.036
		Right	4.5892 (4.59)		
Filial	NAcc to post insula	Left	2.2943 (5.33)	−2.045	0.041
		Right	3.4782 (13.47)		

#### 3.3.3. Relationship between CPI and reward responsiveness

**Figure 6.**
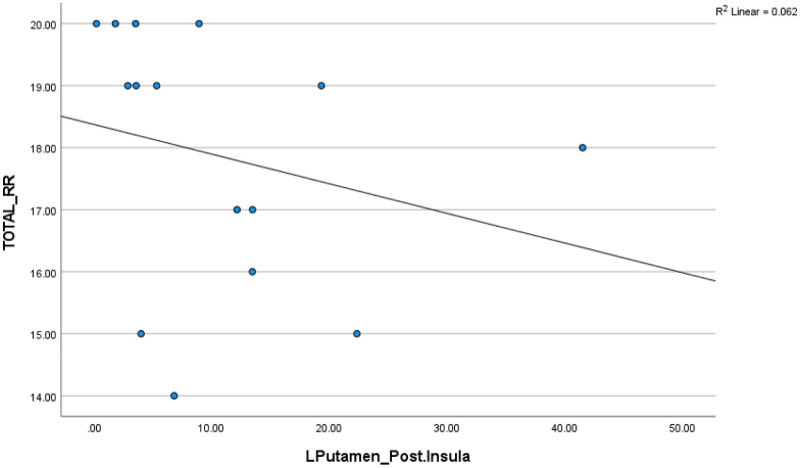
Correlation between total reward responsiveness (RR) and CPI from the left putamen to the posterior insula in the cash group (r = −0.55).

**Figure 7.**
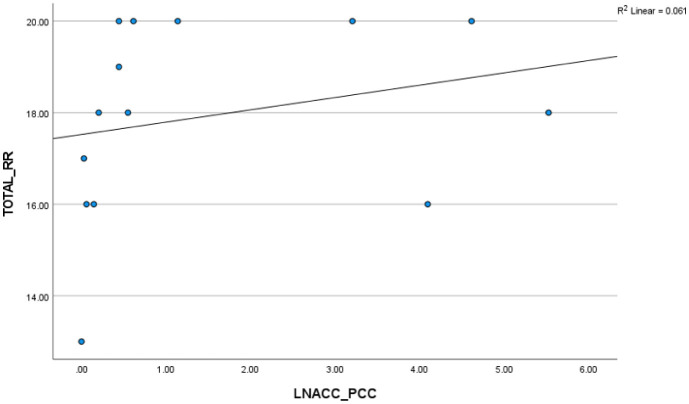
Correlation between total reward responsiveness (RR) and CPI from the left NAcc to the PCC in the filial group (r = 0.55).

In the cash group, a significant negative correlation was found between the CPI from the left putamen to the posterior insula and the total reward responsiveness, r_s_ (13) = −0.55, p < 0.05 (Figure 6). This indicates that higher reward responsiveness is associated with reduced white matter connectivity between the left putamen and posterior insula in the cash group. Conversely, in the filial group, there was a positive correlation between the CPI from the left NAcc to PCC and the total reward responsiveness, r_s_ (12) = 0.55, p < 0.05 (Figure 7). This suggests that higher reward responsiveness leads to stronger white matter connectivity between the left NAcc and PCC in the filial group.

## 4. Discussion

Our study showed that monetary rewards, whether directed at oneself or parents, were perceived as more motivating than non-monetary rewards in this young adult population, as evidenced by better cognitive performance. The n-back task used focuses on working memory, so the differences likely reflected differences in working memory capacity or differences in reward effort, i.e., the cognitive function of the participants, not just the reward cue preference alone. To minimize these differences, we took into consideration the educational background of the participants and only recruited participants from a tertiary academic background with a limited age range. A meta-analysis on 51 studies on the effect of reward on working memory found solid evidence that reward improves working memory performance [47], hence giving credibility to our paradigm of using reward cues prior to the task. Previous research has also established monetary rewards as a powerful motivator and commonly used benchmark in neuroimaging [48],[49] and EEG [50] studies. However, altruistic rewards, such as for parents, appear to fulfil social and belonging needs [51], particularly in populations with strong parental bonds [52].

While previous research showed that the NAcc is typically activated by monetary rewards [22],[53]–[55], we observed no significant activation in this region in the present study. This is possibly due to individual differences in reward sensitivity and task design. Most previous research used monetary incentive delay (MID) tasks, which included both anticipation and receipt phases [53],[56], whereas the current study only included the anticipation phase without feedback. The absence of a receipt phase may have contributed to reduced NAcc activation, as this region is often engaged during the processing of reward outcomes. The differential engagement of striatal subregions, putamen (action-oriented) and NAcc (motivational), likely reflects the specific demands of the task paradigm that placed relatively greater demands on working memory and action execution, rather than pure reward anticipation. This cognitive-motor emphasis may preferentially engage the dorsal striatum (putamen) over the ventral striatum (NAcc).

Our study showed that, in the cash group, the contrast cash > filial cues activated the putamen, a region involved with reward-driven behaviors contingent on specific actions rather than mere anticipation [57]. While the activation of the putamen did not survive the correction threshold and should be interpreted with caution, the result was consistent with prior studies implicating its role in reward processing [20],[21],[24]; the reporting of this finding is still informative. This activation may reflect the strategic thinking involved in selecting rewards, as the putamen supports “hot” executive functions that are essential for reward processing, delay discounting, emotion regulation, and risky decision-making, as well as motor function related to movement initiation and control [58]–[61]. An addiction study by Brewer et al. [62] found that while the NAcc is involved in craving, the putamen is associated with the experience of a “rush”, further supporting its role in action-driven reward experiences. Additionally, reduced putamen activity has been associated with amotivation in depressed patients, suggesting that it may play a role in maintaining motivational states [63]. While the NAcc releases dopamine in response to rewards, the putamen helps regulate dopamine for movement control [60],[61], a function that may explain the activation seen here, as participants engaged in strategic decision-making during the task. Ren et al. [58] highlighted the putamen's critical role in supporting optimal risky decision-making, particularly in age-related cognitive processes.

Our findings revealed greater white matter connectivity between the left NAcc and PCC associated with higher reward responsiveness in the filial group. Importantly, our findings agree with the theories of PCC in social cognition, i.e., self-referential processing and familiarity [62]. PCC, as part of the default mode network (DMN), is known for its involvement in attention, autobiographical memory, and conscious awareness [33], as well as studies indicating that familiar faces activate the PCC [31]. Our previous research showed enhanced pain tolerance and intrinsic connectivity in the ACC-PCC-MCC circuit when participants were accompanied by a loved one [32].

Our findings also revealed lateralized white matter connectivity differences between cash and filial groups. In the cash group, connectivity from the right putamen to the ACC was stronger than the filial group, suggesting heightened engagement in reward evaluation and decision-making [58],[63] in those with a preference for getting rewards for themselves compared to those choosing to give a reward to their parents. Given the involvement of the putamen in reward processing and the ACC in evaluating rewards, it is plausible that the neural circuits connecting these regions may exhibit differential activation or connectivity patterns based on the type of reward being processed.

Higher white matter connectivity was also observed between the left putamen and DLPFC in the cash group. Previous evidence of lateralization in reward processing has stemmed primarily from functional neuroimaging studies. Hu et al. [64] showed that an activation of the left putamen is positively linked with reward sensitivity, with greater activation seen when expectations are high. Soutschek & Tobler [65] found that disrupting the left DLPFC activation via transcranial direct current stimulation (tDCS) reduced motivation and slowed responses during effort exertion. The DLPFC, a region associated with “cold” executive functions [59], is critical for goal-directed planning and execution [66]. Impaired left DLPFC function is linked to reduced motivation for achieving valued outcomes [66]. Thus, the higher white matter connectivity between the left putamen and DLPFC in the cash group suggests a heightened level of motivation and effort dedicated to obtaining the desired monetary reward for oneself. Conversely, the filial group did not show this lateralization.

Another notable lateralization in white matter connectivity was observed in the cash group, with higher connectivity between the left NAcc and ACC compared to the right hemisphere. Both regions are involved in reward-related decision-making: the NAcc detects reward cues, the ACC monitors conflicting situations, and together with the orbitofrontal cortex (OFC), they support goal-directed behaviors [67]. While some functional imaging studies found no hemispheric difference in the NAcc–ACC circuit during monetary reward processing [50], others have noted right lateralization [25]. Although we observed left lateralization connectivity in the cash group, it is possible that third regions or distinct task demands mediate this connectivity, as functional connections do not always align with anatomical pathways [25].

In contrast, the filial group demonstrated lateralization to the right insula, with activation for the contrast filial > cash cues and filial > certificate cues, as well as stronger structural connectivity between right NAcc and right posterior insula. The right anterior insula plays a key role in interoception, integrating bodily states with emotional and motivational processes [68]. Studies have shown that the posterior insula is involved in processing sensory information, while the anterior insula integrates these signals with emotional and cognitive data [69]. Our findings may reflect the filial group's emotional and motivational response to the filial cue, which may have been processed differently in the right hemisphere.

The reward responsiveness subscale reflects an individual's positive response to rewards. In the cash group, a negative correlation between the left putamen and the posterior insula connectivity and reward responsiveness scores suggests that individuals favoring self-reward showed lower connectivity in this pathway with higher reward responsiveness. Studies have reported both positive and negative correlations between reward responsiveness and white matter connectivity [10],[70],[71]. One interpretation of this negative correlation is that individuals with higher susceptibility to motivational stimuli may show reduced connectivity between reward processing regions, potentially reflecting a higher sensitivity to immediate rewards or emotional cues [10].

Our study has several limitations. With only 15–16 participants per group, the study may lack power to detect subtle effects. Additionally, we were unable to control for individual variations in perception of reward value, and the duration of the reward cue used in our paradigm may not be enough to fully explore the neural signals due to the perception of reward. We report the fMRI uncorrected results as exploratory observations, pending confirmation in future studies with larger sample sizes and refined stimulation timing. As a cross-sectional study, it means that factors such as mood or health at the time of testing could influence task performance, impacting neural outcomes. Future research could also explore these findings longitudinally to determine how neural connectivity patterns change with evolving socioeconomic or psychological factors, as well as variations in reward magnitude by post-scan ratings of reward value or desirability. The task design of future studies should also include both anticipation and feedback phases to ensure the capturing of NAcc involvement that is strongly implicated in the receipt phase of reward.

## 5. Conclusions

This study explored the neural mechanisms of self and filial rewards, revealing distinct brain activation patterns and white matter connectivity associated with each type of reward. Our findings revealed that monetary rewards to the self activated traditional reward circuitry, while filial rewards engaged regions linked to autobiographical memory and interoception.

## Use of AI tools declaration

The authors declare they have not used Artificial Intelligence (AI) tools in the creation of this article.
